# Effects of Growth Regulators and Gelling Agents on Ex Vitro Rooting of Raspberry

**DOI:** 10.3390/plants8010003

**Published:** 2018-12-22

**Authors:** Vadim Lebedev, Mikhail Arkaev, Mariya Dremova, Ivan Pozdniakov, Konstantin Shestibratov

**Affiliations:** 1 Pushchino State Institute of Natural Sciences, Prospekt Nauki 3, Pushchino, Moscow Region 142290, Russia; arkmihey@mail.ru (M.A.); marusya.mida@yandex.ru (M.D.); 2 Branch of the Shemyakin-Ovchinnikov Institute of Bioorganic Chemistry of the Russian Academy of Sciences, Prospekt Nauki 6, Pushchino, Moscow Region 142290, Russia; schestibratov.k@yandex.ru; 3 ООО Microklon, P.O. Box 1671, Pushchino, Moscow Region 142290, Russia; zamdir@microklon.ru

**Keywords:** *Rubus idaeus*, acclimatization, ex vitro rooting, plant-growth regulators, Phytagel

## Abstract

Successful acclimatization and ex vitro rooting are among the key factors reducing the cost of micropropagated plants. We compared the survival of seven Russian cultivars of raspberry (*Rubus idaeus*) after rooting in vitro and ex vitro. Rooted shoots adapted to nonsterile conditions much better than nonrooted ones, with survival rates of 81%–98% versus 43%–76%, respectively. We studied the effects of different combinations of plant-growth regulators and gelling agents added to a proliferation medium on ex vitro rooting of primocane-fruiting raspberry cultivar “Atlant”. Reducing the agar concentration from 8 to 6.5 g/L increased the multiplication rate, but caused shoot hyperhydricity. The highest survival rate (97.2%) was observed for shoots grown in a medium containing 0.2 and 0.1 mg/L IBA, and gelled with 5 g/L agar and 0.2 g/L Phytagel. The microshoot height at the multiplication stage did not correlate with the plant growth during acclimatization. The obtained results can be used in the commercial micropropagation of the raspberry.

## 1. Introduction

The raspberry (*Rubus idaeus* L.) belongs to the *Rosaceae* family and is one of the most important berry cultures worldwide. Raspberries are popular due to their appealing color and delicious taste. They also have high nutritional value as they are rich in polyphenols known for their high antioxidant activity and other biologically active compounds [1]. Traditionally, the commercial propagation of raspberry plants is done vegetatively, using cuttings, root suckers, or layering [2]. However, these conventional techniques are time-consuming and do not yield virus-free planting material. Clonal micropropagation allows both the elimination of viruses and the establishment of rapidly multiplying uniform high-quality plants [3]. Micropropagated raspberry plants are used for mother stock plant establishment as well as for the creation of commercial berry plantations. Studies have shown that in vitro propagated raspberry plants under field conditions are phenotypically similar to those propagated by traditional techniques, and even demonstrate better winter hardiness, fruit yield, and weight [4,5]. Moreover, various assays demonstrated the lack of genetic changes in plants produced via tissue culture and their suitability for commercial use [6].

Studies on the in vitro propagation of *Rubus* species started in the 1970s [7]. Although the main efforts were focused on raspberry (*R. idaeus* L.), blackberry (*R. fruticosus* L.), and hybrids between them, micropropagation of other *Rubus* species was also a subject of study [8,9,10]. Researchers investigated the effects of different factors, such as pretreatment and the culture-initiation stage [11], plant-growth regulators [12,13], and the mineral composition of the medium [14] on raspberry micropropagation using most modern analytical methods, such as a metabolomics assay [15]. A critical step in the micropropagation of *Rubus* species is the acclimatization of plantlets to ex vitro conditions; therefore, it is very important to improve the efficiency of this stage [2]. Artificial fog is most commonly used for plantlet acclimatization, although sometimes other methods are applied, e.g., float hydroculture [16].

Another critical stage is in vitro rooting. As the process is very expensive (accounts for 35%–75% of the micropropagation cost), laboratories often try to eliminate the stage by using ex vitro rooting, i.e., by simultaneous plant rooting and acclimatization [17]. The technique not only reduces the cost but also considerably shortens the time of plant production and improves their survival [18]. However, it requires the development of special protocols. Studies of ex vitro rooting have long focused on optimizing the auxin pulse treatment of unrooted microshoots, primarily those of woody plants [19,20,21]. In recent years, however, there has been increasing interest to other factors involved in the process. The effects of different substrates [22] and lighting conditions [17] were assessed after transferring plantlets to a nonsterile environment. The effects of cytokinins [19], the number of subcultures [23], light spectrum [24], and carbon-source type and concentration [25] on the subsequent ex vitro rooting were assessed during the multiplication stage. However, we do not know of any studies on the aftereffects of gelling agents contained in a shoot-proliferation medium on the subsequent ex vitro rooting of the microcuttings.

The objective of the present study was to assess the acclimatization of rooted and unrooted shoots of various Russian raspberry cultivars using a method earlier developed in our laboratory [26], and to evaluate the effects of the types and concentrations of plant-growth regulators and gelling agents in a proliferation medium on ex vitro rooting of the primocane-fruiting raspberry cultivar “Atlant”. To our knowledge, this is the first report describing the effects of gelling agents on ex vitro rooting.

## 2. Results

**Figure 1:**
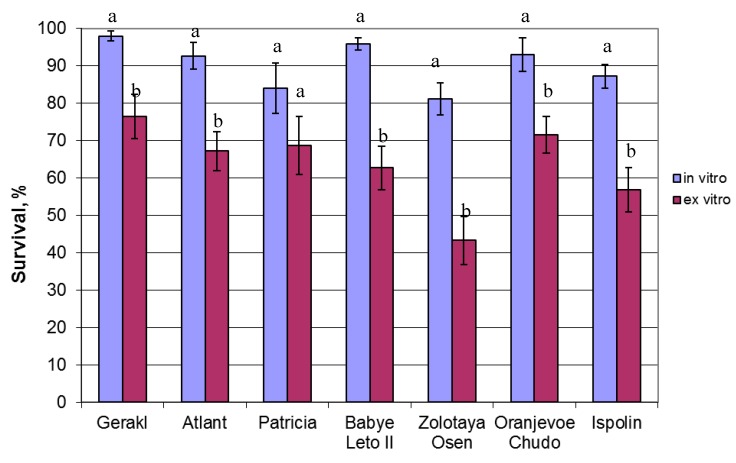
Survival of rooted (in vitro) and unrooted (ex vitro) raspberry shoots in the greenhouse. Different letters indicate significant difference between the rooting methods (*p* < 0.05).

The experiment demonstrated a significant effect of the raspberry genotype on acclimatization (*p* = 0.113 and 0.182 for in vitro and ex vitro rooting, respectively). The survival of in vitro rooted plants varied from 97.9% (“Gerakl”) to 81.1% (“Zolotaya Osen”) (Figure 1), but leaf chlorosis was observed among cultivars. In the case of ex vitro rooting (use of unrooted microcuttings), survival rate was notably lower, ranging from 43.3% to 76.4%. The differences in survival rate between the two rooting methods were significant for most of the cultivars, with the exception of “Patricia”.

**Figure 2:**
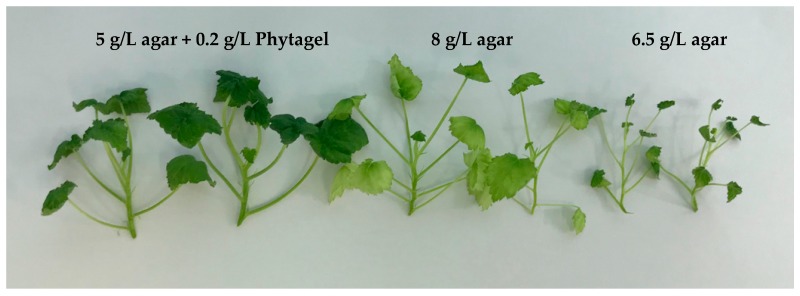
Effect of gelling agents on the physiological status of the “Atlant” raspberry: normal at 5 g/L agar + 0.2 g/L Phytagel (left); chlorosis at 8 g/L agar (center); hyperhydricity at 6.5 g/L agar (right).

The “Atlant” cultivar demonstrated moderate survival after ex vitro rooting and leaf chlorosis during the acclimatization of both rooted and unrooted shoots; the cultivar was used to evaluate the effect of proliferation conditions on the quality of microshoots. Growth regulators and gelling agents had a significant effect on cultivar multiplication. The multiplication rate varied from 2.62 to 4.19 and tended to increase on the media containing 6.5 g/L agar, and to decrease in the media with 8g/L agar. As shown by statistical analysis, growth regulators substantially (*p* = 0.006) influenced plant height, with the maximum height being achieved with BA (0.2 or 0.5 mg/L) + 0.1 mg/L IBA (Table 1). The effect of gelling agents on the quantitative parameters (height) was insignificant (*p* = 0.874), yet very important for shoot quality: hyperhydricity of microshoots was observed for all treatments with 6.5 g/L agar, and leaf chlorosis was seen in some treatments with 8 g/L agar (Table 1, Figure 2). In addition, plants showed spontaneous root formation in the media with 6.5 g/L agar.

Changes in the composition of the proliferation medium significantly affected the quality of the shoots that were subsequently used for ex vitro rooting. On the whole, the survival of the “Atlant” plants after their ex vitro rooting improved compared to the previous value of 71.4% obtained for shoots grown on the medium with 0.7 mg/L BA and 8 g/L agar (Figure 1), although a decrease was observed in some treatments (media with 6.5 g/L agar) (Table 2). The highest survival rate was in shoots grown in the medium containing 0.2 mg/L BA + 0.1 mg/L IBA and gelled with 5 g/L agar + 0.2 mg/L Phytagel. Shoots grown in the medium with agar and Phytagel and containing 0.1 mg/L BA + 0.5 mg/L kinetin demonstrated the best combination of survival and growth.

**Figure 3:**
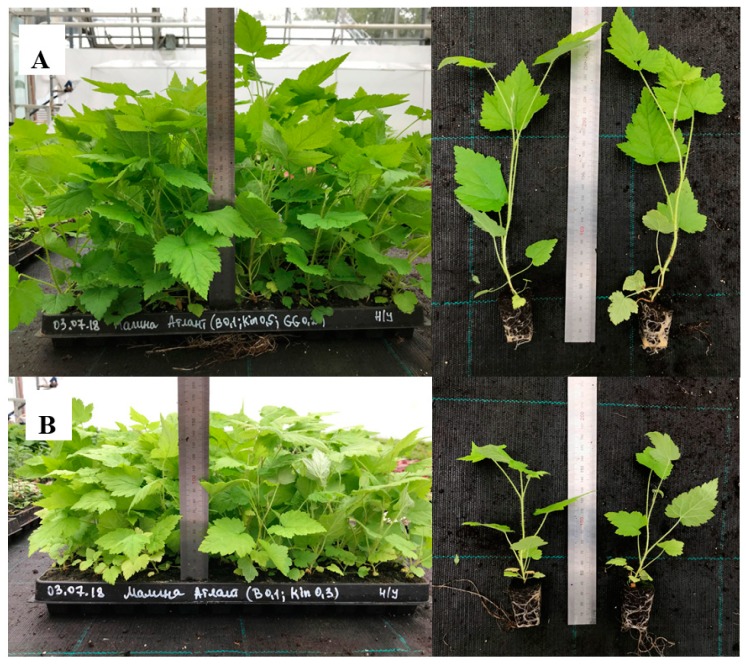
Ex vitro rooting of the “Atlant” raspberry. (**A**) after multiplication on the medium with 0.1 mg/L BA, 0.5 mg/L kinetin, 5 g/L agar, 0.2 g/L Phytagel; (**B**) after 0.1 mg/L BA, 0.3 mg/L kinetin, 8 g/L agar.

Survival rate was significantly affected by the gelling agent and its interaction with the growth regulators, but never by the growth regulators alone (Table 3). However, height was influenced by each of the factors as well by their interaction. There were great differences in the height of the survived plants after six weeks of acclimatization, with their height ranging from 54.1 to 125.5 mm (Table 2, Figure 3). The tallest plants were obtained after proliferation in media with 8 g/L agar, while the shortest ones after proliferation in media with 6.5 g/L agar (Table 2). No correlation was found between plant height at the multiplication stage and their height after the acclimatization (correlation coefficient = 0.053). At the same time, we observed correlation between the height of acclimatized plants and their physiological state at the proliferation stage: hyperhydricity and chlorosis impeded plant growth (Table 1 and Table 2).

The best treatment was the medium supplemented with 0.1 mg/L BA + 0.5 mg/L kinetin and 5 g/L agar + 0.2 mg/L Phytagel. After proliferation on this medium, unrooted microshoots showed optimal combination of survival and growth in the greenhouse. Shoots grown in the medium with 0.2 mg/L BA + 0.1 mg/L IBA, 5 g/L agar + 0.2 mg/L Phytagel demonstrated a slightly better survival rate but were considerably shorter. Shoot growth in media containing 8 g/L agar (0.1 mg/L BA + 0.5 mg/L kinetin, 0.5 mg/L BA + 0.1 mg/L IBA) was equally good, but survival rate was significantly lower (Table 2). In addition, a high multiplication rate was observed on the medium.

## 3. Discussion

Micropropagation of small-fruit species and vegetative rootstocks is the most successful commercial application of in vitro culture for fruit crops [6]. One of the key reasons is that it makes it possible to obtain virus-free planting material and, thus, significantly increase crop productivity. The main obstacle to a large-scale use of the clonal micropropagation method is the high mortality rate of plants during their acclimatization to non-sterile conditions [27]. We had earlier developed a simple method for the acclimatization of in vitro plants using capillary mats and a double-layer covering (a vapor permeable layer and a vapor proof one). The efficiency of the method was demonstrated on forest-tree species: their acclimatization rate reached 96%–100% for common ash [26] and birch plants [28]. Good results were obtained with the rooted microshoots of Russian raspberry cultivars, the acclimatization rate being 81%–98% (Figure 1), which is in agreement with the data of other researchers. For instance, studies of 26 raspberry cultivars showed 73%–100% plant survival after the acclimatization of in vitro rooted plants [29]. However, in vitro rooting considerably increases the cost and time of obtaining micropropagated plants. The ex vitro rooting technique (simultaneous rooting and acclimatization of unrooted microshoots) improves micropropagation efficiency due to time and cost reduction and a simpler process; it also eliminates the risk of root damage during transplantation of rooted plants [30,31]. For instance, ex vitro rooting reduced the cost of micropropagated tea plants by 71% compared with conventional in vitro rooting [32]. The use of this technology reduced the time of in vitro culture for Kopyor coconut plants from 10 to 4 months [33]. However, the survival of unrooted raspberry microshoots obtained under standard multiplication conditions (0.7 mg/L BA and 8 g/L agar) was significantly lower than of those rooted in vitro, 43%–76% versus 81%–98%, respectively (Figure 1). For further experiments aiming at optimizing the multiplication conditions, we chose the new cultivar, “Atlant”, patented in 2015. This cultivar of primocane-fruiting raspberry features high productivity, large berries, and an extended storage period [34].

The conditions of plant micropropagation are known to affect their acclimatization to an ex vitro environment, e.g., substitution of BA by meta-topolin in a proliferation medium improved the acclimatization of rooted shoots of *Uniola paniculata* [35]. The effect of the proliferation medium is even more pronounced for ex vitro rooting because shoots are transferred to nonsterile conditions immediately after the multiplication stage. In our study, we assessed the effect of plant-growth regulators and gelling agents on the subsequent ex vitro rooting of raspberry microcuttings. Multiplication was done in an MS medium, the most suitable medium for raspberry according to multiple studies [29]. We did not use TDZ for shoot proliferation because, along with increasing the multiplication rate, the cytokinin causes unwanted effects in plants [36]. This was also demonstrated on the raspberry, where TDZ induced the formation of deformed, partially hyperhydric, large-leaved shoots [37]. For the proliferation stage, it is important to select a proper concentration of BA. With higher BA concentrations, shoot size decreased and the risk of somaclonal variation increased. Therefore, we used decreased BA concentration and added kinetin in some treatments. The average multiplication rate after 4 weeks of culture was about 3.4 and depended both on growth regulators and on the gelling agent. Height, however, did not depend on the gelling agent, but the reduction of agar content from 8 to 6.5 g/L caused the hyperhydricity of the shoots. Therefore, the agar concentration of 6.5 g/L was unsuitable for raspberry micropropagation as it led to hyperhydricity [38]. At the same time, no hyperhydricity was observed in the medium with 5 g/L agar and 0.2 g/L Phytagel. It can be assumed that 0.2 g/L Phytagel compensated for the decrease in agar concentration.

It is known that raspberry shoots in vitro suffer from leaf chlorosis [39]. The main cause of chlorosis in vitro is iron deficiency. To prevent chlorosis, we doubled the concentration of iron in the nutrient media. Even so, there were some yellowing leaves on the acclimatized raspberry plants of the “Atlant” cultivar. We think this could be due to the cultivar’s particular sensitivity to auxins in the rooting medium, which are known to be capable of inducing leaf chlorosis upon long exposure [40]. The yellowing shoots on the proliferation medium with 8 g/L agar (Table 1) could be a genotypic reaction to the type and concentration of agar because no chlorosis was observed in other treatments. It is known that some types of agar can cause the chlorosis of shoots [41]. In addition, the agar concentration of 8 g/L agar might have been excessive for raspberry proliferation since the multiplication rate at this concentration was the lowest (Table 1). High agar concentrations can reduce the growth rate [38].

The statistical analysis of our data did not show any specific effect of growth regulators on the survival of raspberry plants during ex vitro rooting (Table 3). Similarly, cytokinins in the proliferation medium showed no effect on the ex vitro rooting of pistachios [19] and blueberries [24]. However, reducing the BA concentration and modifying the content of gelling agents as important components of the proliferation medium compared with initial composition (0.7 mg/L BA, 8 g/L agar) significantly improved the efficiency of ex vitro rooting of the raspberry, which, in some treatments, exceeded 90% (Table 2). Carbohydrates, another important component of the nutrient medium, had a notable effect on the ex vitro rooting of banana plants [25]. We do not know of any studies on the role of different gelling agents in the micropropagation of the raspberry, but complete or partial substitution of agar by Phytagel had no significant impact on shoot or callus regeneration from blackberry leaves [42]. We demonstrated that raspberry microcuttings most successfully rooted ex vitro if grown on a medium gelled with a combination of agar and Phytagel. According to Kumar and Palni [43], rose plants cultured in Phytagel showed better survival and growth in a greenhouse than those cultured in agar. Ismail et al. [41] demonstrated that a combination of Gelrite and agar in the culture medium facilitated optimal multiplication of high-quality acacia shoots. The best combination of plant survival and growth after ex vitro rooting was observed for raspberry shoots that had proliferated in the medium with 0.1 mg/L BA and 0.5 mg/L kinetin. This treatment also resulted in one of the highest multiplication rates (Table 1), which is the main economic aspect in the commercial micropropagation of the raspberry [37]. We obtained lower survival rates after multiplication in media containing 6.5 g/L agar that caused shoot hyperhydricity. The acclimatized plants grown in these media were the shortest ones. Although, according to some reports [44], when transferred to soil, hyperhydric plants grew faster than the normal ones, hyperhydricity is generally considered to be a physiological abnormality.

It is noteworthy that we obtained high survival rates in the summertime. Meanwhile, it is known that acclimatization to ex vitro conditions should be done taking the season into account because high midsummer temperatures reduce the viability of plants [2]. This is in contrast to a report by Clapa et al. [37] where unrooted raspberry shoots were unable to acclimatize in the greenhouse, whereas we achieved high efficiency of ex vitro rooting. Shoot height at the multiplication stage had no effect on their growth under nonsterile conditions, which suggests that plant acclimatization was more dependent on shoot quality rather than on size. In our study, chlorotic shoots grew slower than nonchlorotic ones in the greenhouse. The plants with higher chlorophyll content may have higher chances for survival and better growth during acclimatization [45]. Leaf color as an indicator of chlorophyll content can be used for the diagnosis of plant nutritional status. In the future, we plan to evaluate the responses of different raspberry cultivars to in vitro and ex vitro conditions using nondestructive image-based phenotyping technologies.

## 4. Materials and Methods 

### 4.1. Plant Material and Culture Conditions

The following Russian raspberry cultivars were used: “Atlant”, “Babye Leto II”, “Gerakl”, “Zolotaya Osen”, “Ispolin”, “Oranjevoe Chudo”, and “Patricia”. Multiplication was carried out in the MS medium [46] containing mineral salts and vitamins, with double Fe concentration and 0.7 mg/L BA. Explants were transferred to a fresh medium every 4 weeks. The shoots were rooted in the QL medium [47] with half macrosalt content, 0.1 mg/L IBA, and 0.1 mg/L IAA for 3 weeks. All nutrient media contained 20 g/L sucrose and 8 g/L agar. The media were sterilized by autoclaving at 121 °C for 20 min. Plant-growth regulators and vitamins were sterilized by filtration (0.22 μm Millipore filters) and added to the media after autoclaving. The plants were cultured at 22–24 °C, 16/8 h photoperiod. 

### 4.2. Acclimatization

Acclimatization of raspberry microshoots was performed according to an earlier developed protocol [26]. Both the unrooted shoots after the proliferation stage (ex vitro rooting) and the rooted shoots after the rooting stage (in vitro rooting) were used for all cultivars. The shoots were planted in a greenhouse in plastic seedling trays (144 cells, 24 ml cell volume) with a substrate containing peat and perlite in a 3:1 ratio. The trays were cut into 36-cell sections, randomly located on the greenhouse shelving (each treatment consisted of 4 replicates; 144 shoots per treatment). The trays with the plants were placed onto capillary mats and covered with a layer of spunbond and a layer of polyethylene film for two weeks (90%–95% humidity). After that, the film layer was removed and the plants were maintained under the spunbond layer during one week (70%–75% humidity). Plant survival and height were assessed 6 weeks after planting the shoots in the greenhouse.

### 4.3. Ex Vitro Rooting

For the ex vitro rooting experiment, “Atlant” microshoots were proliferated in an MS medium. A total of 12 treatments were used: 4 combinations of plant-growth regulators (0.1 mg/L BA + 0.5 mg/L kinetin, 0.1 mg/L BA + 0.3 mg/L kinetin, 0.2 mg/L BA + 0.1 mg/L IBA, and 0.2 mg/L BA + 0.1 mg/L IBA) and 3 combinations of gelling agents (8 g/L agar, 6.5 g/L agar, and 5 g/L agar + 0.2 g/L Phytagel (Sigma)). The shoots were multiplied in 300 ml glass jars containing 12 explants each (20–25 jars per treatment). Multiplication rate, shoot height, and physiological disorders (hyperhydricity, chlorosis) were estimated after 4 weeks in 6 randomly selected jars per treatment. Unrooted raspberry microcuttings, about 20 mm in length, were used for acclimatization, as described above. Each treatment consisted of 4 replicates (144-cell trays) arranged in a completely randomized design. Survival equates to rooting as all unrooted shoots died within 6 weeks under nonsterile conditions.

### 4.4. Statistical Analysis

Statistical treatment of the data was performed by analysis of variance (ANOVA) using Statistica 10 software (StatSoft, Tulsa, OK, USA). Percentage data were transformed to arcsine values prior to analysis. Genotype ranking was carried out according to the Duncan test: genotypes marked by the same letters had no reliable difference with 95% probability. The experiments were repeated twice.

## 5. Conclusions

In this paper, we reported the ex vitro rooting of the raspberry that can be used to reduce the cost and labor intensity of plant micropropagation. Unrooted microshoots of primocane-fruiting raspberry cultivar “Atlant” were simultaneously rooted and acclimatized in the greenhouse with up to 97.2% survival rate. The best results were obtained for shoots grown in a medium gelled with agar and supplemented with Phytagel. Reducing agar concentration in the multiplication medium caused hyperhydricity of shoots, worsened their acclimatization, and retarded their growth. The described protocol can be successfully used for the accelerated commercial propagation of the raspberry.

## Figures and Tables

**Table 1:** Effect of growth regulators and gelling agents on the multiplication of the “Atlant” raspberry.

Gelling Agent	Growth Regulators (mg/L)	Multiplication Rate	Height (mm)	Hyperhydricity	Chlorosis
Agar 8 g/L	BA 0.1 + kin 0.5	3.14 ± 0.17 bcd ^1^	22.5 ± 0.4 abc	-	-
	BA 0.1 + kin 0.3	2.62 ± 0.19 d	21.6 ± 0.9 abcd	-	+
	BA 0.2 + IBA 0.1	3.21 ± 0.22 bcd	19.3 ± 1.1 cd	-	+
	BA 0.5 + IBA 0.1	2.98 ± 0.16 cd	22.3 ± 2.3 abc	-	-
Agar 6.5 g/L	BA 0.1 + kin 0.5	4.19 ± 0.19 a	21.4 ± 1.8 bcd	+	-
	BA 0.1 + kin 0.3	3.74 ± 0.26 ab	18.2 ± 1.6 cd	+	-
	BA 0.2 + IBA 0.1	2.90 ± 0.15 cd	19.1 ± 1.2 cd	+	-
	BA 0.5 + IBA 0.1	3.31 ± 0.14 bc	25.1 ± 1.7 ab	+	-
Agar 5 g/L +	BA 0.1 + kin 0.5	3.64 ± 0.24 ab	20.1 ± 1.6 cd	-	-
Phytagel 0.2 g/L	BA 0.1 + kin 0.3	3.31 ± 0.20 bc	17.0 ± 1.3 d	-	-
	BA 0.2 + IBA 0.1	3.36 ± 0.17 bc	26.1 ± 1.4 a	-	-
	BA 0.5 + IBA 0.1	3.00 ± 0.13 cd	22.4 ± 0.7 abc	-	-

^1^ Different letters indicate significant differences between treatments (*p* < 0.05).

**Table 2:** Effect of proliferation medium composition on subsequent ex vitro rooting of the “Atlant” raspberry.

Gelling Agent	Growth Regulators (mg/L)	Survival (%)	Height (mm)
Agar 8 g/L	BA 0.1 + kin 0.5	74.3 ± 3.7 de ^1^	125.5 ± 8.9 a
	BA 0.1 + kin 0.3	89.9 ± 3.8 bc	76.1 ± 6.0 cde
	BA 0.2 + IBA 0.1	80.2 ± 3.1 cde	73.1 ± 5.6 de
	BA 0.5 + IBA 0.1	77.5 ± 4.7 cde	118.0 ± 7.9 a
Agar 6.5 g/L	BA 0.1 + kin 0.5	83.9 ± 3.5 bcd	65.6 ± 3.5 ef
	BA 0.1 + kin 0.3	85.4 ± 3.5 bcd	88.2 ± 4.7 bcd
	BA 0.2 + IBA 0.1	65.6 ± 4.7 ef	70.3 ± 4.2 def
	BA 0.5 + IBA 0.1	60.9 ± 2.9 f	54.1 ± 4.5 f
Agar 5 g/L +	BA 0.1 + kin 0.5	91.6 ± 3.6 ab	117.4 ± 7.2 a
Phytagel 0.2 g/L	BA 0.1 + kin. 0.3	78.8 ± 4.4 cde	99.5 ± 2.9 b
	BA 0.2 + IBA 0.1	97.2 ± 2.0 a	85.8 ± 3.4 bcd
	BA 0.5 + IBA 0.1	82.2 ± 5.4 bcd	93.6 ± 6.9 bc

^1^ Different letters indicate significant differences between treatments (*p* < 0.05).

**Table 3:** Analysis of variance of ex vitro rooting of the “Atlant” raspberry.

Factors	Degrees of	Survival		Shoot Height	
	Freedom	F-Value	*p*	F-Value	*p*
Growth regulators	3	2.805	0.053471	10.543	0.000041
Gelling agent	2	12.376	0.000081	33.845	0.000000
Growth regulator x gelling agent	6	6.926	0.000060	11.771	0.000000
Error	36				

## References

[1] Lee J., Dossett M., Finn C.E. (2012). *Rubus* fruit phenolic research: The food, the bad, and the confusing. Food Chem..

[2] Dziedzic E., Jagła J., Lambardi M., Ozudogru E.A., Jain S.M. (2013). Micropropagation of *Rubus* and *Ribes* spp.. Protocols for Micropropagation of Selected Economically Important Horticultural Plants.

[3] Martin R.R. (2002). Virus diseases of *Rubus* and strategies for their control. Acta Hortic..

[4] Bite A., Petrevica L. (2002). The influence of *in vitro* propagation on the field behaviour of red raspberry variety ‘Norna’. Acta Hortic..

[5] Georgieva M., Kondakova V., Dragoyski K., Georgiev D., Naydenova G. (2009). Comparative study of raspberry cv. Balgarski Rubin propagated by classical and *in vitro* methods. J. Pomol..

[6] Vujović T., Ružić D., Cerović R., Leposavić A., Karaklajić-Stajić Z., Mitrović O., Žurawicz E. (2017). An assessment of the genetic integrity of micropropagated raspberry and blackberry plants. Sci. Hortic..

[7] Snir I., Bajaj Y.P.S. (1988). Red Raspberry (*Rubus idaeus*). Crops II. Biotechnology in Agriculture and Forestry.

[8] Martinussen I., Nilsen G., Svenson L., Junttila O., Rapp K. (2004). In vitro propagation of cloudberry (*Rubus chamaemorus*). Plant Cell Tissue Organ Cult..

[9] Najaf-Abadi A., Jafari Hamidoghli Y. (2009). Micropropagation of thornless trailing blackberry (*Rubus* sp.) by axillary bud explants. Aust. J. Crop Sci..

[10] Ismaini L., Destri Surya M.I. (2017). Micropropagation of *Rubus chrysophyllus* Reinw. ex Miq. and *Rubus fraxinifolius* Poir. J. Trop. Life Sci..

[11] Wu J.H., Miller S.A., Hall H.K., Mooney P.A. (2009). Factors affecting the efficiency of micropropagation from lateral buds and shoot tips of *Rubus*. Plant Cell Tissus Organ Cult..

[12] Gonzales M.V., Lopez M., Valdes A.E., Ordas R.J. (2000). Micropropagation of three berry fruit species using nodal segments from field—Grown plants. Ann. Appl. Biol..

[13] Hunkova J., Libiakova G., Gaidosova A. (2016). Shoot proliferation ability of selected cultivars of *Rubus* spp. as influenced by genotype and cytokinin concentration. J. Cent. Eur. Agric..

[14] Poothong S., Reed B.M. (2014). Modeling the effects of mineral nutrition for improving growth and development of micropropagated red raspberries. Sci. Hortic..

[15] Poothong S., Morré J., Maier C.S., Reed B.M. (2017). Metabolic changes and improved growth in micropropagated red raspberry “Indian summer” are tied to improved mineral nutrition. In Vitro Cell. Dev. Biol. Plant.

[16] Clapa D., Fira A., Joshee N. (2013). An efficient *ex vitro* rooting and acclimatization method for horticultural plants using float hydroculture. Hortscience.

[17] Wozny A., Miler N. (2016). LEDs application in ex vitro rooting and acclimatization of chrysanthemum (*Chrysanthemum* x *grandiflorum*/Ramat./Kitam). Electron. J. Pol. Agric. Univ..

[18] Singh A., Agarwal P.K. (2016). Enhanced micropropagation protocol of ex vitro rooting of a commercially important crop plant *Simmondsia chinensis* (Link) Schneider. J. For. Sci..

[19] Benmahioul B., Dorion N., Kaid-Harche M., Daguin F. (2012). Micropropagation and ex vitro rooting of pistachio (*Pistacia vera* L.). Plant Cell Tissue Organ Cult..

[20] Aygun A., Dumanoglu H. (2015). In vitro shoot proliferation and in vitro and ex vitro root formation of *Pyrus elaeagrifolia* Pallas. Front. Plant Sci..

[21] Shekafandeh A., Shahcheraghi S.T. (2017). Ex vitro rooting and survival of regenerated shoots ‎from three fig (*Ficus carica* L.) genotypes. Agric. Conspec. Sci..

[22] Clapa D., Fira A., Simu M. (2015). The role of rooting substrate in blackberry ex vitro rooting and acclimatization stage. ProEnviron. Promediu.

[23] Leva A. (2011). Innovative protocol for ‘ex vitro rooting’ on olive micropropagation. Cent. Eur. J. Biol..

[24] Hung C.D., Hong C.H., Kim S.K., Lee K.H., Park J.Y., Nam M.W., Choi D.H., Lee H.I. (2016). LED light for in vitro and ex vitro efficient growth of economically important highbush blueberry (*Vaccinium corymbosum* L.). Acta Physiol. Plant..

[25] Bohra P., Waman A.A., Sathyanarayana B.N., Umesha K. (2016). Concurrent ex vitro rooting and hardening in Ney Poovan Banana (*Musa* AB): Effect of carbon sources and their concentrations. Erwerbs-Obstbau.

[26] Lebedev V., Shestibratov K. (2016). Large-scale micropropagation of common ash. Biotechnology.

[27] Kumar K., Rao U. (2012). Morphophysiological problems in acclimatization of micropropagated plants in—ex vitro conditions—A reviews. J. Ornam. Hortic. Plants.

[28] Lebedev V.G., Azarova A.B., Arkaev M.S., Nevskii S.A., Shestibratov K.A. (2017). Effective mass propagation of various *Betula* species via in vitro culturing. Biotekhnologiya.

[29] Isac V., Popescu A., Mezzetti B., Ružić Đ., Gajdosova A. (2009). Protocol for in vitro micropropagation of raspberry and plant regeneration by organogenesis. A Guide to Some In Vitro Techniques—Small Fruits.

[30] Sharma U., Kataria V., Shekhawat N.S. (2017). In vitro propagation, ex vitro rooting and leaf micromorphology of *Bauhinia racemosa* Lam.: A leguminous tree with medicinal values. Physiol. Mol. Biol. Plants.

[31] Shekhawat M.S., Manokari M. (2018). In vitro multiplication, micromorphological studies and ex vitro rooting of *Hybanthus enneaspermus* (L.) F. Muell.—A rare medicinal plant. Acta Bot. Croat..

[32] Ranaweera K.K., Gunasekara M.T.K., Eeswara J.P. (2013). Ex vitro rooting: A low cost micropropagation technique for tea (*Camellia sinensis* (L.) O. Kuntz hybrids. Sci. Hortic..

[33] Sisunandar A., Husin A., Julianto T., Yuniaty A., Rival A., Adkins S. (2018). Ex vitro rooting using a mini growth chamber increases root induction and accelerates acclimatization of Kopyor coconut (*Cocos nucifera* L.) embryo culture-derived seedlings. In Vitro Cell. Dev. Biol. Plant.

[34] Hummer K., Hall H.K., Funt R.C., Hall H.K. (2013). Raspberries. Raspberries. Crop Production Science in Horticulture.

[35] Valero-Aracama C., Kane M.E., Wilson S.B., Philman N.L. (2010). Substitution of benzyladenine with meta-topolin during shoot multiplication increases acclimatization of difficult- and easy-to-acclimatize sea oats (*Uniola*
*paniculata* L.) genotypes. Plant Growth Regul..

[36] Lu C.-Y. (1993). The use of thidiazuron in tissue culture. In Vitro Cell. Dev. Biol. Plant.

[37] Clapa D., Fira A., Pacurar I. (2008). The in vitro propagation of the raspberry cultivar *Citria*. Bull. UASMV.

[38] Thorpe T.A., Stasolla C., Yeung E.C., de Klerk G.-J., Roberts A., George E.F., George E.F., Hall M.A., de Klerk G.-J. (2008). The components of plant tissue culture media II: Organic additions, osmotic and pH effects, and support systems. Plant Propagation by Tissue Culture.

[39] Zawadzka M., Orlikowska T. (2006). The influence of FeEDDHA in red raspberry cultures during shoot multiplication and adventitious regeneration from leaf explants. Plant Cell Tissue Organ Cult..

[40] Bhojwani S.S., Dantu P.K., Bhojwani S.S., Dantu P.K. (2013). Micropropagation. Plant Tissue Culture: An Introductory Text.

[41] Ismail H., Kumar S.M., Aziah M.Y., Hasnida N.H., Nor Aini A.S. (2016). In vitro micropropagation of *Acacia auriculiformis* from selected juvenile sources. Dendrobiology.

[42] Tsao C.W.V., Reed B.M. (2002). Gelling agents, silver nitrate, and sequestrene iron influence adventitious shoot and callus formation from *Rubus* leaves. In Vitro Cell. Dev. Biol. Plant.

[43] Kumar A., Palni L.M.S. (2003). The effect of light source and gelling agent on micropropagation of *Rosa damascena* Mill. and *Rhynchostylis retusa* (L.) Bl. J. Hortic. Sci. Biotechnol..

[44] Dries N.V., Gianni S., Czerednik A., Krens FA., Klerk G.M. (2013). Flooding of the apoplast is a key factor in the development of hyperhydricity. J. Exp. Bot..

[45] Christensen B., Sriskandarajah S., Serek M., Muller R. (2008). In vitro culture of *Hibiscus rosa-sinensis* L.: Influence of iron, calcium and BAP on establishment and multiplication. Plant Cell Tissue Organ Cult..

[46] Murashige T., Skoog F. (1962). A revised medium for rapid growth and bioassay of tobacco tissue cultures. Physiol. Plant.

[47] Quoirin M., Lepoivre P. (1977). Improved media for *in vitro* culture of *Prunus* species. Acta Hortic..

